# Tabby graphene: Dimensional magnetic crossover in fluorinated graphite

**DOI:** 10.1038/s41598-017-16321-5

**Published:** 2017-11-29

**Authors:** T. L. Makarova, A. L. Shelankov, A. I. Shames, A. A. Zyrianova, A. A. Komlev, G. N. Chekhova, D. V. Pinakov, L. G. Bulusheva, A. V. Okotrub, E. Lähderanta

**Affiliations:** 1 Lappeenranta University of Technology, Lappeenranta, 53851 Finland; 2 Ioffe Institute, St. Petersburg, 194021 Russian Federation; 3 Ben-Gurion University of the Negev, Be’er-Sheva, 8410501 Israel; 4 St. Petersburg State University, St. Petersburg, 199034 Russian Federation; 5 Nikolaev Institute of Inorganic Chemistry SB RAS, Novosibirsk, 630090 Russian Federation; 6 Novosibirsk State University, Novosibirsk, 630090 Russian Federation

## Abstract

*Tabby* is a pattern of short irregular stripes, usually related to domestic cats. We have produced *Tabby* patterns on graphene by attaching fluorine atoms running as monoatomic chains in crystallographic directions. Separated by non-fluorinated *sp* ^2^ carbon ribbons, *sp* ^3^-hybridized carbon atoms bonded to zigzag fluorine chains produce *sp* ^2^-*sp* ^3^ interfaces and spin-polarized edge states localized on both sides of the chains. We have compared two kinds of fluorinated graphite samples C_2_F_*x*_, with *x* near to 1 and *x* substantially below 1. The magnetic susceptibility of C_2_F_*x*_ (*x* < 1) shows a broad maximum and a thermally activated spin gap behaviour that can be understood in a two-leg spin ladder model with ferromagnetic legs and antiferromagnetic rungs; the spin gap constitutes about 450 K. Besides, stable room-temperature ferromagnetism is observed in C_2_F_*x*_ (*x* < 1) samples: the crossover to a three-dimensional magnetic behaviour is due to the onset of interlayer interactions. Similarly prepared C_2_F_*x*_ (*x* ≈ 1) samples demonstrate features of two-dimensional magnetism without signs of high-temperature magnetic ordering, but with transition to a superparamagnetic state below 40 K instead. The magnetism of the *Tabby* graphene is stable until 520 K, which is the temperature of the structural reconstruction of fluorinated graphite.

## Introduction

Magnetism in restricted dimensions can be studied in real bulk crystals if the exchange interactions are much stronger in one or two spatial directions than in the remaining ones^1,2^. Thus, low dimension magnets have the advantage of bulk materials in providing sufficient intensity for experiments investigating the thermodynamic and spectroscopic characterization of magnetism. Most studies of low dimensional magnetism concentrate on molecular magnets based on organic radicals (see e.g.^3,4^) or Cu and Ni compounds which have spins ½ or 1, correspondingly^5^. We have recently synthesized a novel graphene derivative decorated by monoatomic fluorine chains running in crystallographic directions, and have observed clear signs of one-dimensional-like magnetism in this two-dimensional material^6^. Nanoscale magnetic activity of pure graphene is controlled by the edge geometry. In the present study, graphene fluorination, instead of breaking of the carbon-carbon bonds, is used as an efficient approach to generate edges and, therefore, correlated magnetic states in this material. The fluorine chain running in zigzag direction induces strong spin polarization with a mixed ferro-antiferro-magnetic coupling between locally emerged magnetic moments.

**Figure 1:**
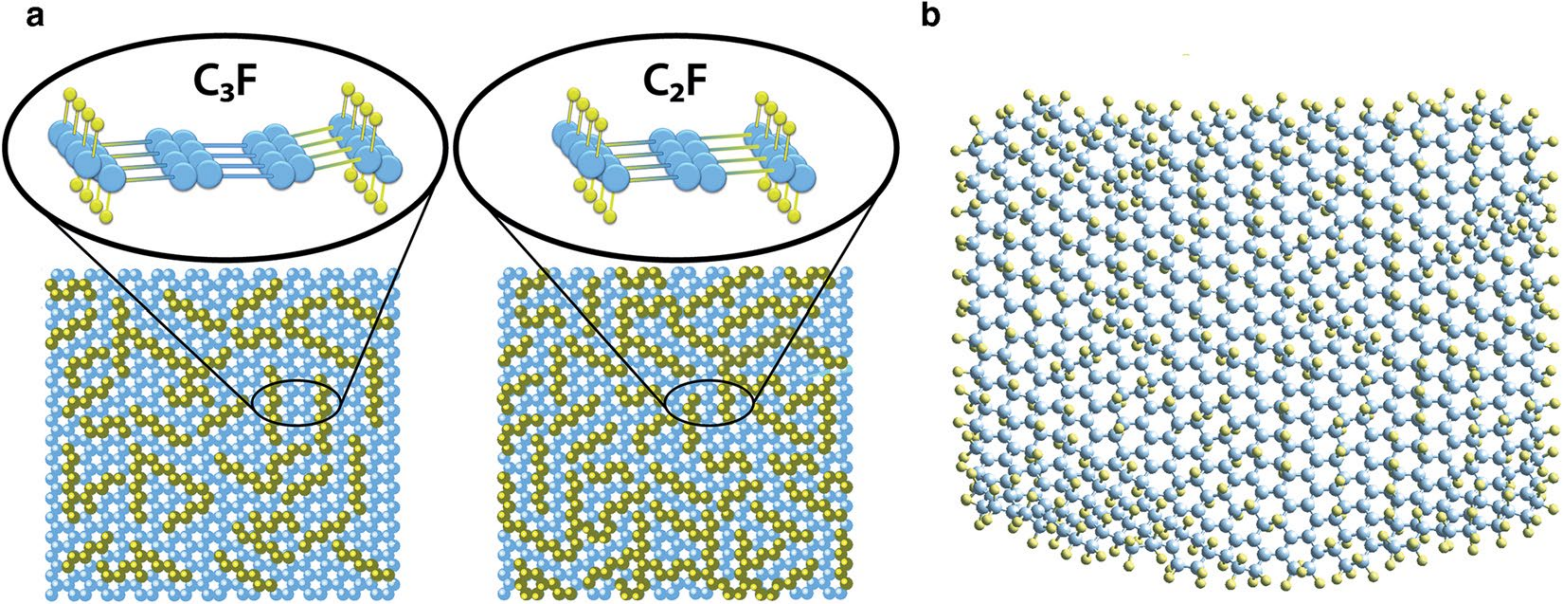
Basal plane of deficiently fluorinated graphene: (**a**) Embedded nanoribbons; (**b**) *Tabby* pattern is plotted according to the results of spectroscopic investigations^7,28,29^. The blue circles denote carbon atoms, and the yellow circles denote fluorine atoms.

A distinctive characteristic of the novel derivative is that the interfaces form *Tabby* stripes. *Tabby* is a pattern of a cat’s coat with tiger stripes and leopard spots. Ideally, if the *sp* ^2^-*sp* ^3^ interfaces were parallel and evenly spaced lines, they would form embedded graphene nanoribbons. The stoichiometry C_4_F yields a 3-carbon atom wide nanoribbon, C_3_F is a 2-atom wide one, and C_2_F produces monoatomic chains (Fig. 1a). However, this ideal picture is only partially applicable, because the stripes run in all crystallographic directions (Fig. 1b). Still, the *Tabby* graphene is a unique derivative because the retention of the π-electron system results in different electronic properties of the *Tabby* graphene compared to fully functionalized graphene derivatives, which are insulators. The *Tabby* graphene contains confined islands of the π-electron system, it is semiconducting with the band gap 2–2.5 eV, and colour dependent on the C/F ratio^7^. The most distinct feature is that the *Tabby* graphene contains up to 10% of stable unpaired spins on carbon atoms. These states interact antiferromagnetically or ferromagnetically, depending on sublattice position.

We implemented the zigzag edge states at *sp* ^2^-*sp* ^3^ interfaces by slow fluorination of graphite intercalation compounds, which expanded into graphenes during the synthesis. This approach allowed us to obtain single- or bi-layers of fluorinated graphenes. In the case of the fully fluorinated graphene CF, all π-electrons on the basal planes were spent for covalent bonding with fluorine^8^, whereas deficient fluorination (Fig. 1) preserved the π-system partially, and its electronic properties were sensitive to the fluorine coverage^9,10^. Spin-half Curie paramagnetism in graphene functionalized with fluorine has been observed previously^11^. Quite recently, a ferromagnetic-like response has been found in hydroxofluorographenes and interpreted as due to biradical states^12^.

In this paper, we report on data demonstrating that the π-electron network in carbon nanosegments formed by *Tabby* fluorine patterns on the basal planes, shows non-Curie paramagnetism and magnetic order, depending on the stacking of graphitic planes and the fluorine coverage. We concentrate on similarities and differences in the magnetic properties of the *Tabby* graphene for various fluorine contents.

## Results

### Magnetic properties of Tabby graphene C_2_F_*x*_ with *x* < 1

**Figure 2:**
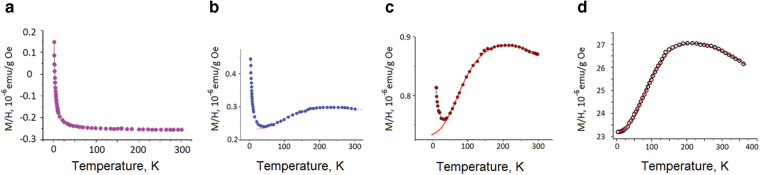
Evolution of magnetic susceptibility *vs*. temperature upon ageing of C_2_F_*x*_ (*x* < 1): (**a**) A Curie-like spin magnetism observed in as prepared samples; (**b**) and (**c**) progressive changes after few months of storage; (**d**) after 1 year.

The magnetic properties of *Tabby* graphenes are tightly connected with their structure, which changes during the sample history. Immediately after the synthesis, the samples were diamagnetic with only a Curie-like paramagnetic tail at low temperatures^13^. Unexpectedly, the measurements performed in several months, during which the samples were stored in a desiccator at room temperature, revealed qualitative changes: in addition to spin-half paramagnetism (Fig. 2a), the magnetic susceptibility as a function of temperature exhibited a maximum at ~250 K (Fig. 2b). The contribution of this non-Curie paramagnetism increased during further ageing (Fig. 2c), and in about one year the broad maximum followed by an activation-like drop on cooling (Fig. 2d) became a dominating feature in the magnetic susceptibility. Remarkably, the magnetization increased by 50 times after one year of storage.

The linear magnetic susceptibility χ(*T*) in Fig. 2 can be split as χ = χ_0_ + χ_Curie_ + χ_spin_ into a temperature-independent term χ_0_, a small Curie-like part χ_Curie_ = C/*T* identified by the low-*T* upturn, and a spin contribution χ_spin_. The term χ_spin_(*T*), that is the nontrivial part of the magnetic response, shows a non-monotonous temperature dependence with a broad maximum. This feature *cannot* be understood in the frames of independent magnetic moments. As shown in Fig. 2, non-monotonous temperature dependence of magnetic susceptibility χ_spin_ is the hallmark of low dimensional magnetism and is typical for both chain- and ladder-structured materials^14^. The position of the maximum in the plot of χ_spin_ (*T*) establishes an energy scale related to the strength of antiferromagnetic (AF) spin-spin interaction which favours spin pairing, and therefore, opposes the spin alignment parallel to an external magnetic field^15^.

To extract the strength of the interaction from the data and to estimate the amount of interacting spins, one may think in terms of localized spins with the Heisenberg Hamiltonian, H = Σ_ij_ *J*_ij_ ***S***_i_·***S***_j_ where ***S***_i_ is the spin located at site i and *J*_ij_ denotes the strength of the exchange interaction. As in the case of molecule-based materials, various models can be tried out: the Heisenberg chain with anisotropic antiferromagnetic coupling (Bonner-Fisher^16^), the Hatfield model^17^ of a 1D spin chain with modulated coupling, a dimerised chain (Bleaney-Bowers^18^), and a spin ladder with antiferromagnetic interactions (Troyer-Tsunetsugu-Würtz^19^). However, none of these models which assume various modifications of only antiferromagnetic couplings, could give satisfactory agreement with our experiment. Indeed, the picture of the spin interactions that was predicted by the discoverers of the peculiar edge states of zigzag graphene nanoribbons in the seminal work by Wakabayashi *et al*.^20^, is characterized by ferromagnetic exchange coupling *J*_FM_ ~ 10^3^ K within a zigzag edge and an antiferromagnetic edge-edge coupling of the order *J*_AFM_ ~ 10–100 K, depending on the nanoribbon width^21^. It is therefore reasonable to interpret the experimental results in the frames of the spin ladder model with ferromagnetic legs (*J*_FM_) and antiferromagnetic rungs (*J*_AFM_). We calculated the magnetic susceptibility of this model for the number of spins in a leg, N_s_, changing from 1 to 12^6^, and found that this model could be used successfully to fit a large batch of samples. As an example, the solid line in Fig. 2d corresponds to a combination of N_s_ = 3 and N_s_ = 8.

On the basis of the analysis of the susceptibility curves obtained on the different samples, we have arrived at the following quantitative conclusion: (i) with the maximum at *T* = 200–280 K, the exchange interaction strength (*J*_AFM_) value is in the range *J*_AFM = _300–450 K^22^; (ii) the concentration of the exchange-coupled spins is rather large, being in the range 1–20% per C_n_F structural unit; (iii) the residual concentration of isolated spins ½ does not exceed 0.1%. The *J*_AFM_ value confirms quantitatively that the AFM interactions within the graphene planes are much stronger than those in the organic radical crystals (e.g. nitroxide-based radicals, verdazyl radical crystals, or thiazyl radicals^3^), making the *Tabby* graphene an ideal candidate for 2D behaviour studies of pure organic materials.

### Evolution of the magnetism of C_2_F_*x*_ (*x* < 1) on ageing and annealing

**Figure 3:**
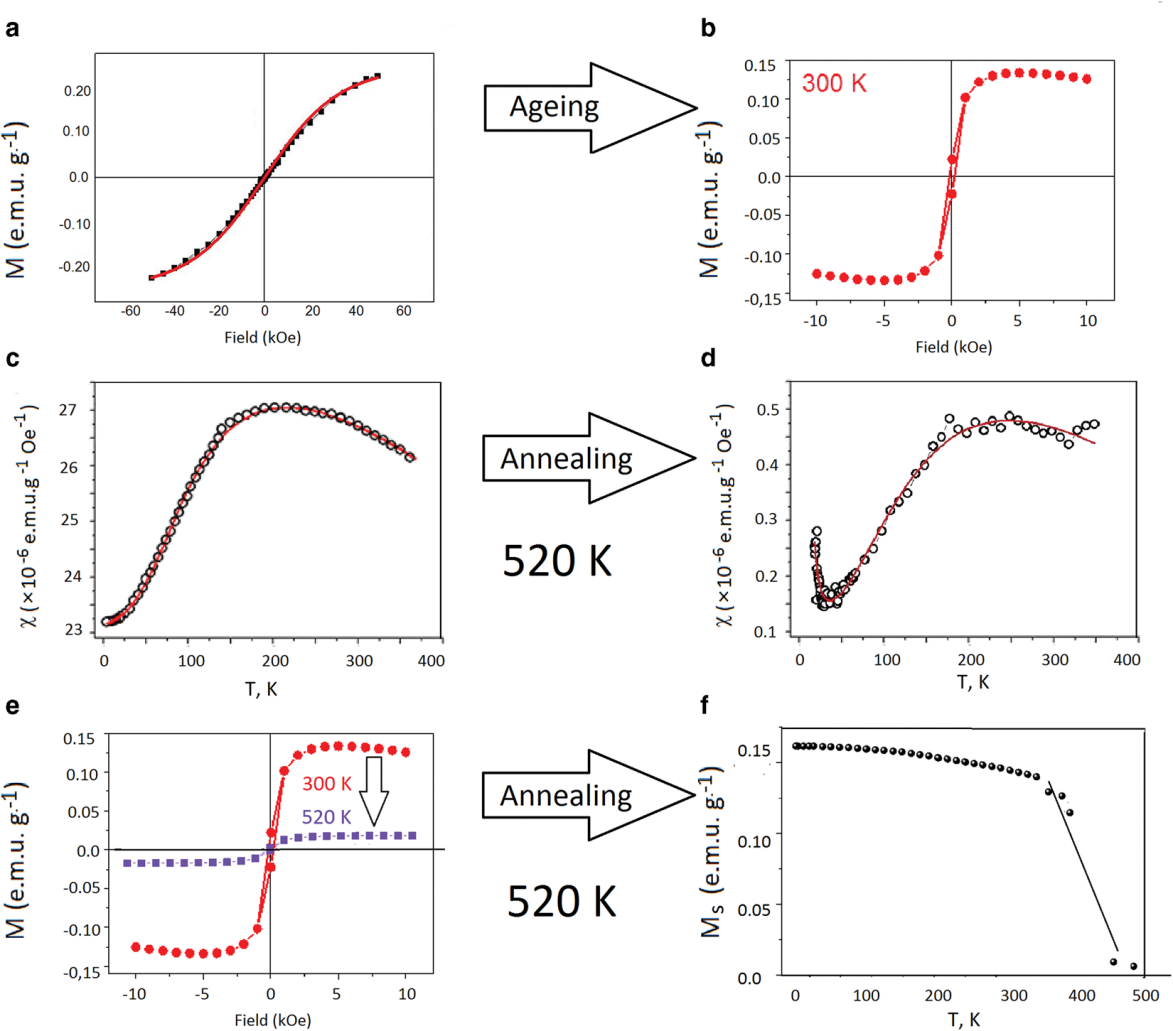
Evolution of the ferromagnetic properties of the *Tabby* graphene C_2_F_*x*_ (*x* < 1) upon ageing and annealing: (**a**) magnetic moment M as a function of a magnetic field *H*; the symbols are the measurements and the solid curve fits the Brillouin function with *S* = ½ and *g* = 2; (**b**) M(*H*) dependence of the same sample after 1 year of ageing at room temperature. The temperature dependences of the magnetic susceptibility of the aged sample; (**c**) the same sample after short-term heating to the fluorine detachment temperature 520 K (**d**). M(*H*) dependence of an aged sample and the same sample after short-term heating to 520 K (**e**). Temperature dependence of the saturation magnetization M_s_ for the *Tabby* graphene; the sharp decrease of M_s_ around 400 K is due to the beginning of thermal irreversible destruction of the sample (**f**).

A remarkable property of the *Tabby* graphene is the development of macroscopic magnetic order during ageing. Figure 3a shows that the behaviour of as-prepared samples is accurately described by the Brillouin function, which provides a good fit for total angular momentum quantum number *J* = *S* = ½ (free isolated electron spin). The saturation value in the magnetization curve shown in Fig. 3b corresponds to 0.3% of localized spins participating in the long-range magnetic order.

As the samples were purely organic, their magnetism was prone to thermal effects. We observed a sharp drop of paramagnetic response (Fig. 3c,d) and saturated magnetization (Fig. 3e) as a result of short-term heating up to the fluorine detachment temperature (520 K). This confirms that the magnetism of the *Tabby* graphene has pure organic origin and is determined by the fluorine arrangement on the basal plane.

We made an attempt to determine the Curie temperature for this organic magnet by measuring the isothermal M(*H*) loops and plotting the saturated magnetization at each temperature point (Fig. 3f). The Curie temperature was higher than the room temperature, but its exact value could not be determined because the magnetism falled irreversibly due to thermal destruction of the samples.

### Magnetic properties of C_2_F_*x*_ with *x ≈* 1

**Figure 4:**
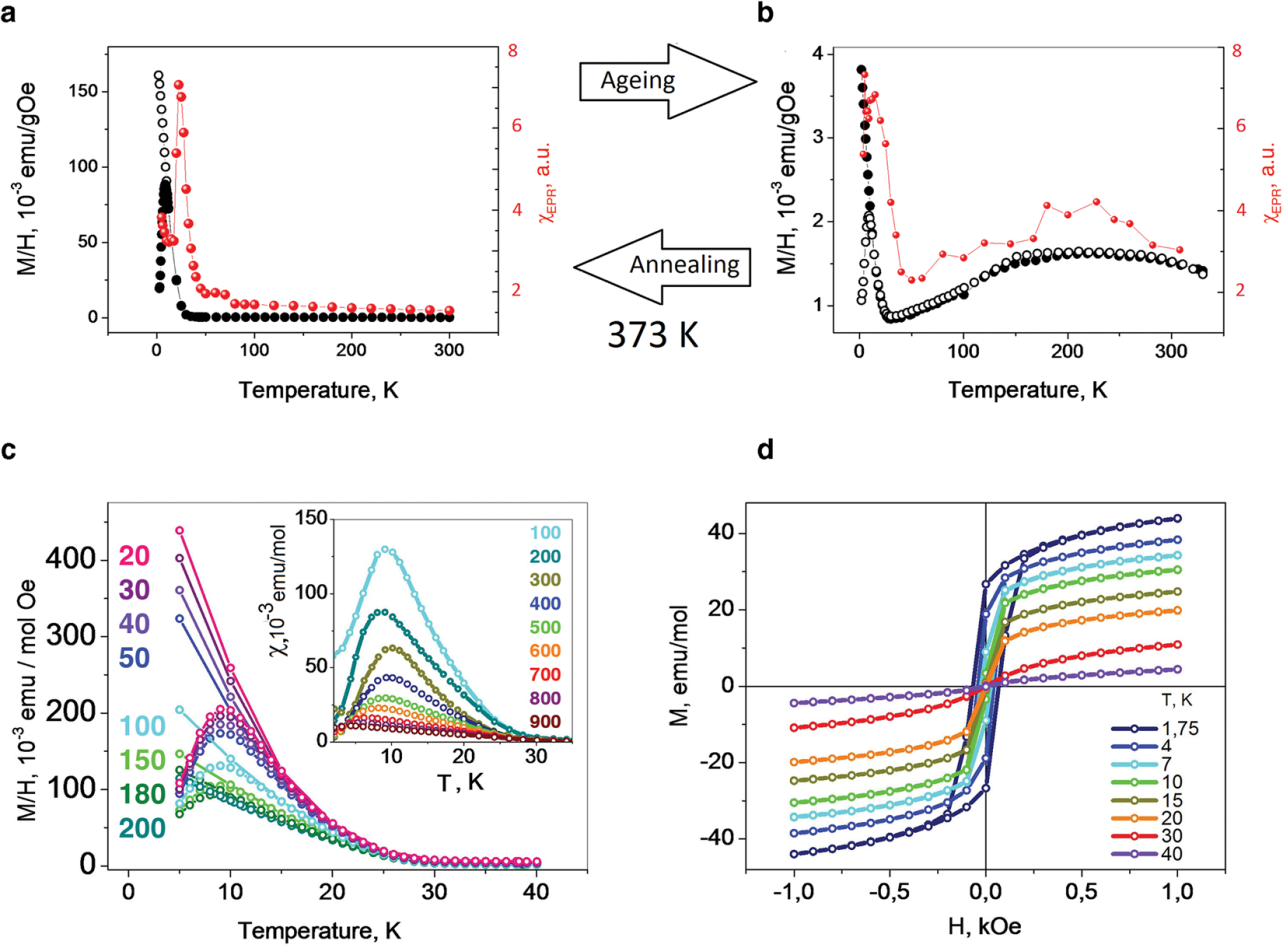
Temperature dependencies of magnetic susceptibility for *Tabby* graphenes, C_2_F_*x*_ (*x* *≈* 1*)*: (**a**) pristine samples; (**b**) aged samples – the open circles represent the ZFC, the solid circles are the FC measurements, the red symbols represent the double integrated intensity (the EPR susceptibility); (**c**) M(*T*) curves taken at different fields; (**d**) M(*H*) dependencies taken at different temperatures.

The *Tabby* graphenes of the composition C_2_F_*x*_ (*x* *≈* 1) were prepared by using similar technological procedures as in the case of C_2_F_*x*_, x < 1 (see the supplementary information for more details). All samples were Curie-like paramagnetic at room temperatures without any sign of ferromagnetism. However, on cooling, a strong increase in the magnetic moment was observed, pointing out at a ferromagnetic-like transition near 40 K (Fig. 4a). This transition was registered by both Superconducting Quantum Interference Device (SQUID) and Electron Paramagnetic Resonance (EPR). The double integrated intensity (EPR susceptibility) followed the ZFC magnetization protocols. The zero field–cooled (ZFC) and the field-cooled (FC) magnetizations diverged below 16 K, indicating a slow relaxation (blocking) of magnetization (Fig. 4a). This low-temperature behaviour was sensitive to the applied magnetic fields (*H*), and at *H* = 1000 Oe the difference between the ZFC and FC magnetizations was undetectable (Fig. 4c). The position of the shifts to lower temperatures as the field increased, yielding the anisotropy field *H*_A_ = 2600 Oe and a zero-field value of the blocking temperature, *T* ^0^_max_ = 10.5 K. M(*H*) isothermal dependencies (Fig. 4d), confirmed that below 40 K we observed superparamagnetism, and the numerical fits of the data in Fig. 4c,d give an average spin quantum number *S* ≅ 1000.

Keeping the samples at room temperature for a year caused strong changes to the magnetic response (Fig. 4b). The magnetism of the aged samples looked similar at first glance, but really it was not. First, the values of magnetic moment below the ferromagnetic-like transition were 40–50 times smaller in the aged samples. Second, on cooling from room temperature, the magnetic susceptibility passed through a maximum around 280 K and approached zero at low temperatures. This indicates clearly that the temperature dependence of magnetic susceptibility exhibits an activated behaviour below a broad maximum, which is characteristic of thermal excitation from a nonmagnetic ground state with a spin gap. Annealing the samples in a dynamic vacuum at *T* = 373 K for 24 hours resulted in full restoration of the magnetic properties shown in Fig. 4a. This resulted in the reduction of the oxygen and water content from 0.47% to 0.19%, and from 1.21% to 0.75% correspondingly, as verified by X-ray photoelectron spectroscopy. This led probably to the increase of interlayer interactions and development of 3D superparamagnetic ordering below 40 K. Apparently, both oxygen and water pushed the graphene planes apart, and the magnetic response was dominated by short-range 2D antiferromagnetic interactions.

### EPR study of C_2_F_*x*_ with *x ≈* 1

The EPR technique, which allows selective tracking of different magnetic entities, diagnoses the magnetic units responsible for the described phenomena. For all the studied samples, the room temperature EPR spectra revealed two main constituents: (a) narrow lines within the region of *g* = 2.00, typical for fluorinated carbon, and (b) asymmetric broad lines with *g* ~ 2. The total spin densities were found to be 10^18^–10^20^ spin/g with only 1–2% contribution coming from the narrow signals. We focused the attention on the broad lines. For all samples, doubly integrated intensities for the broad lines which corresponded to EPR susceptibility followed the trends for SQUID susceptibility (see Fig. 4a,b), while the differences between the FC and ZFC measurements followed the shift of the resonant field positions *H*_r_^broad^. At each temperature, the position of a broad EPR line (i.e., resonant field *H*_r_^broad^) for this paramagnetic entity was determined by the origin of the entities (*g*-factor) and the internal magnetic fields (like hyperfine one etc.). Thus, the developing temperature shift of *H*_r_^broad^ manifested progressive strengthening of the internal magnetic field. It is worth mentioning here that the resonant field of the narrow EPR line, attributed to fluorinated carbon, remained the same within the entire temperature region of 4–300 K. This may have reflected both the inhomogeneity of the samples and the local nature of magnetic ordering in the corresponding entities.

**Figure 5:**
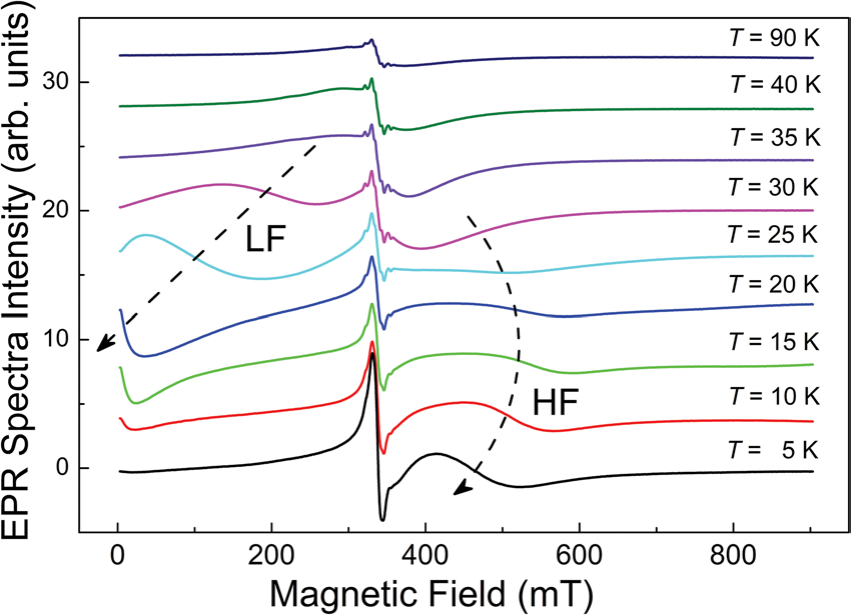
Temperature dependence of EPR spectra for the as prepared *Tabby* graphene, C_2_F_*x*_ (*x* *≈* *1*) at *T* < 100 K. All spectra were recorded at the same experimental conditions: ν = 9.469 GHz, incident microwave power 20 mW, 100 kHz magnetic field modulation amplitude 0.5 mT, and receiver gain 10^4^. The spectra have been shifted vertically for better presentation. The dashed arrows indicate changes in *H*_r_^broad^ for low- and high-field components of the FMR signal at decreasing temperature.

**Figure 6:**
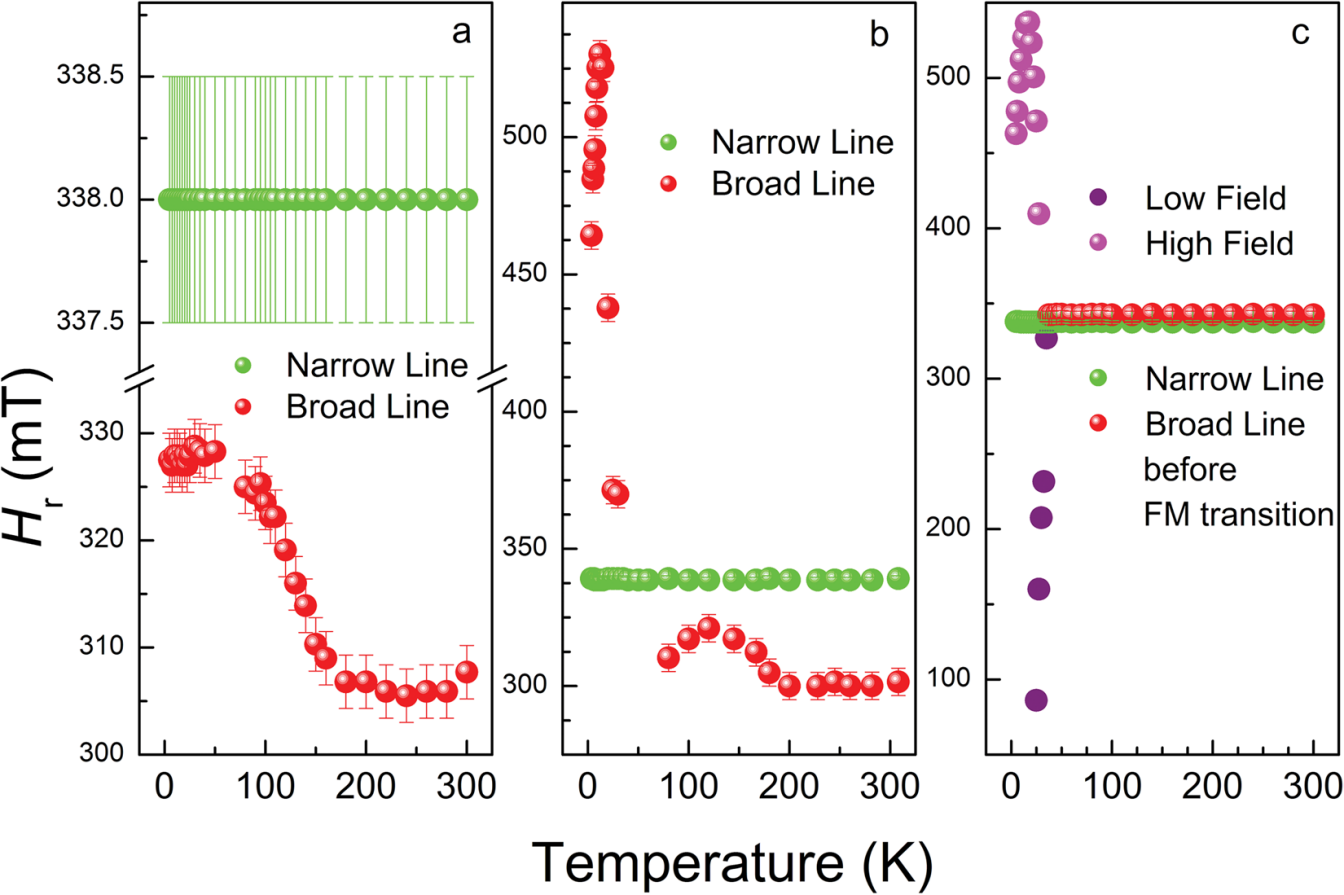
Resonance field positions *H*_r_ of the EPR signal *vs*. temperature for *Tabby* graphene C_2_F_*x*_: (**a**) fluorine content *x* < *1*; (**b**) aged sample with *x* *≈* *1*; and (**c**) the same sample with *x* *≈* *1* as prepared.

An unambiguous attribution of the magnetic unit can be done for as-prepared C_2_F_*x*_ *(x* *≈* 1*)*. There we observed clear transformation of the same broad EPR signal that was observed at room temperatures, into a multicomponent ferromagnetic resonance (FMR) signal (Fig. 5). Below 40 K the broad EPR signal grew abruptly in integral intensity, broadened and then split into low and high field components, which shifted towards opposite directions on further temperature decrease. The low field component kept shifting to the zero field region and then disappeared below 25 K, whereas the high field component at *T* = 15 K turned back to lower resonance field values, still remaining within the high field region (Fig. 6c). All the above features are typical for ferromagnetic (FMR) signals originated from partially oriented ferromagnetic subsystems with different magnetic anisotropies. Thus, the subsystem of exchange-coupled magnetic entities responsible for the short-range in-plane interactions, was also responsible for the ferromagnetism observed in these systems at low temperatures. This subsystem was present in all the studied samples: as-prepared, aged and annealed. Its magnetic behaviour was rich: it demonstrated 2D antiferromagnetic interactions, room temperature magnetic ordering, and the development of low-temperature superparamagnetism. We note that superparamagnetic behaviour below 25 K have been observed in EPR studies of nanosized graphite prepared by ball milling^23^ and of ultrathin graphitic particles obtained by heavy sonication of graphite powder^24^.

In the case of *Tabby* graphenes C_2_F_*x*_ with *x* < 1, the position of the broad line, *H*_r_^broad^, was shifted to the low field region and changed its position smoothly on cooling. This indicates a decrease of an internal spontaneous magnetic field (Fig. 6a). For the case of *Tabby* graphenes with *x* < 1, the same behaviour of *H*_r_^broad^ was observed within the temperature range of 100–300 K. However, below 100 K, the *H*_r_^broad^ in this sample shifted abruptly to the higher field region (Fig. 6b corresponds to the sample depicted in Fig. 4b), indicating significant change in the internal magnetic field on cooling.

## Discussion

Fluorination is well known to be an effective method for introducing localized spin centres into graphene. When the attached fluorine atoms are arranged as monoatomic chains running in crystallographic directions (*Tabby* pattern), the *sp* ^2^-*sp* ^3^ interfaces created by the fluorine chain play the same role as zigzag edges. The graphene bipartite lattice consists of inequivalent A and B sublattices. In the case of a zigzag chain, one expects a set of localized spin states in sublattice A on one side of the nanochain and sublattice B on the other side. Density functional theory (DFT) calculations for well-separated fluorine chains clearly reveal the emergence of magnetism in fluorinated graphene^6^. The calculated complex magnetic configuration combines the strong ferromagnetic interaction between the local magnetic moments of C atoms within each side of the CF chain with the antiferromagnetic coupling between the magnetic moments of C atoms located on the opposite sides of the CF chain.

The parallels in the magnetic behaviour of *Tabby* graphenes with different stoichiometry and stacking sequences lead to a conclusion that the magnetic unit responsible for the ordering effects originates from the π-electron system in the nanosegments created by the *Tabby* patterns with the magnetic moments localized at the zigzag interfaces. DFT calculations confirm that edge states near a single zigzag chain are preserved in disordered networks of densely packed interfaces^6^. Strong differences in the magnetic behaviour of the described samples are explained by their structural differences.

Several graphene derivatives are known, e.g. graphene oxide, hydrogenated graphene, fluorographene, and chlorographene. Functionalization opens the band gap of graphene, which is desirable for on/off electronics. However, at full graphene coverage, the possibilities for manipulation of electronic properties are limited because the π electrons are already spent to the attached atoms. Extensive studies are done on releasing the π electrons by producing single-side C_2_F and C_2_H (still not synthesized), or “digging” the wide-gap derivatives, removing extra atoms and forming one-dimensional graphene regions: point defects, quantum dots, nanoribbons, superlattices.

In the synthesis of the *Tabby* graphene, the method of slow fluorination provides conditions where adsorbates tend to align in a chain sequence in which F adatoms are located on alternating sides of the graphene plane. Fluorine has the highest electronegativity of all elements. If the synthesis is made slowly at low temperature, quasi-equilibrium conditions allow fluorine to attach to the thermodynamically preferable places. Fluorinated carbon tends to separate spatially from non-fluorinated carbon. If one F is attached to the graphene, the next F will most preferably attach close to it, to another side in the ortho-position. The adsorption of an odd number of F atoms disturbs the π-electron system, so the adjacent atom attaches as close as possible, but to another side due to strong repulsion. The meta-position is unfavourable because it creates unpaired radicals.

We observed 2D spin gap -activated magnetism of C_2_F_*x*_ (*x* < 1) samples after prolonged ageing (Fig. 2). It can be speculated that this feature is related to the bilayer-like structure of fluorinated graphite (*x* < 1). During the gas-phase synthesis, simultaneous F adsorption at multiple sites resulted in seeding of the chains in a random distribution across the graphene planes. Apparently, the chains were not long enough to produce the zigzag-inherited edge states. The honeycomb lattice of graphene is a bipartite lattice. Sublattices A and B are identical in a single layer. In a bilayer, atom attachment on B sites is energetically more preferable than on A sites. The chain grown in a certain direction of a graphite layer may dictate the rule for the pattern formation in the adjacent layer. The C−F bonds in graphene are dynamic, with a low energy barrier for the migration of F atom to the nearest C atom. The fluorine chains repeal and arrange in two neighbouring layers like the teeth of a comb. This creates an additional discriminating mechanism leading to the formation of regular *Tabby* patterns where crossing and branching are suppressed, which is presumably favourable for magnetic coupling.

When the layers in the *Tabby* graphene are substantially spaced, as in C_2_F_*x*_ (*x* *≈* 1), the compound can be considered as a stack of monolayers. In this case the discriminating mechanism for chain formation is absent. Although fluorination does produce localized spins, the chain length is not enough for the development of zigzag edge states with measurable magnetic interactions on the 2D plane, or the development of high-temperature long-range magnetic ordering. High-temperature long-range magnetic ordering was absent in the samples of our study. Instead, we observed transition to 3D superparamagnetic behaviour at low temperatures. A low temperature ferromagnetic transition was seen in the multicomponent FMR signal, with the features typical for partially oriented ferromagnetic subsystems having different magnetic anisotropies. Ageing of the samples led to 50-fold reduction in the 3D magnetism, whereas transition to 2D behaviour was detected from the broad maximum and low-temperature activated behaviour of magnetic susceptibility.

To sum up, fluorination of graphene in such a way that the attached fluorine atoms formed monoatomic stripes running in crystallographic directions produced a special type of graphene derivative, which we call the *Tabby* graphene. The *sp* ^2^ *–sp* ^3^ interfaces produced by the fluorine atoms attached in the zigzag directions gave rise to numerous magnetic phenomena. Graphene is a two-dimensional material, whereas the fluorine chain patterns reduce the effective dimensionality to 1D. Magnetic susceptibility shows a behaviour typical for low-dimensional quantum spin-ladder systems, which is characterized by spin ordering along the zigzag edges and their antiparallel alignment between opposite zigzag edges. The existence of a gap in the spin excitation spectra of graphene ribbons with zigzag edges has been theorized since their discovery in 1998. We produced magnetically active fluorinated graphite samples and showed that this type of low-dimensional magnetism can be realized experimentally in a graphene-based system. We observed magnetic dimensional crossover with changes in the fluorine loading and interlayer distance, the parameters that can be controlled by synthesis conditions, sample ageing, and annealing. The *Tabby* graphene is a promising material with tuneable electronic and magnetic properties, and it provides a playground for the exploration of new quantum many-body states as well.

## Methods

### Sample preparation

Samples of fluorinated graphite C_2_F_*x*_ with the fluorine content *x*, 0.5 ≤ *x* ≤ 1, were produced by room temperature synthesis^25–27^. The starting material for the synthesis of the fluorinated graphite samples was natural graphite from the Zaval’evo deposit (Ukraine). To remove metal and silicate impurities, the material was purified by double acid treatment (HNO_3_:HCl 1:3 and concentrated HF). The grain size of the samples was about 100 × 100 × 20 μm, and the content of 3d metal impurities was below 1 ppm. Detailed description of sample preparation can be found in Supplementary Information.

### Characterization methods

DC magnetic measurements were performed at a Quantum Design SQUID magnetometer (MPMS-XL-1) in -1 T–+1 T magnetic fields. The DC magnetic susceptibility data were collected in the 1.76–400 K range in a 10 mT magnetic field. Electron Paramagnetic Resonance (EPR) measurements within the temperature range 4 K < *T* < 300 K were carried out by using a Bruker EMX-200 X-band (ν~9 GHz) EPR spectrometer equipped with Oxford Instrument ESR900 cryostat and Agilent 53150 A frequency counter at microwave power (*P*_MW_) levels ranging from 50 µW to 200 mW. The structure and composition of the fluorinated graphite samples were studied by means of X-ray diffraction (XRD) on a DRON-SEIFERT-RM4 diffractometer by using Cu*K*_*α*_ radiation and X-ray photoelectron spectroscopy (XPS) on a Phoibos 150 SPECS spectrometer using monochromatized Al*K*_*α*_ radiation with the energy of 1486.7 eV. The results of structure and composition analysis are given in Supplementary Information.

### Data availability statement

All data generated or analysed during this study are included in this published article (and its Supplementary Information files).

## Electronic supplementary material

Supplementary Information
