# Supplementary material for: Tabby graphene: Dimensional magnetic crossover in fluorinated graphite
Source: Sci Rep. 2017 Nov 29;7:16544. doi: 10.1038/s41598-017-16321-5 (PMC5707391; doi:10.1038/s41598-017-16321-5)
Supplement: Supplementary file 1 — Supplementary Information [file 41598_2017_16321_MOESM1_ESM.pdf]

SUPPLEMENTARY INFORMATION

Tabby graphene: Dimensional magnetic crossover in fluorinated graphite

T. L. Makarova[a,b†], A. L. Shelankov[b], A. I. Shames[c], A. A. Zyrianova[d], A. A. Komlev[a], G. N. Chekhova[e], D. V. Pinakov[e,f], L. G. Bulusheva[e,f] , A. V. Okotrub[e,f], E. Lähderanta[a*]

[a] *Lappeenranta University of Technology, Lappeenranta, 53851, Finland*

[b] *Ioffe Institute, St. Petersburg, 194021, Russian Federation*

[c] *Ben-Gurion University of the Negev, Be'er-Sheva, 8410501, Israel*

[d] *St. Petersburg State University, St. Petersburg, 199034, Russian Federation*

[e] *Nikolaev Institute of Inorganic Chemistry SB RAS, Novosibirsk, 630090, Russian Federation*

[f] *Novosibirsk State University, Novosibirsk, 630090, Russian Federation*

[†] deceased

[*] Corresponding author. Tel: +358405545227. E-mail: Erkki.Lahderanta@lut.fi (Erkki Lähderanta)

1. Synthesis

A starting material for the synthesis of fluorinated graphite samples was natural graphite from the Zaval'evo deposit (Ukraine). Crystallites with a typical size of 0.10×0.10×0.02 mm were purified from the metal and silicate impurities by double treatment with acids (HNO_3:HCl 1:3 and concentrated HF) and with subsequent annealing at 800°C for 1 h. Optical images of the obtained fluorinated graphite crystallites are shown in Figure S1.

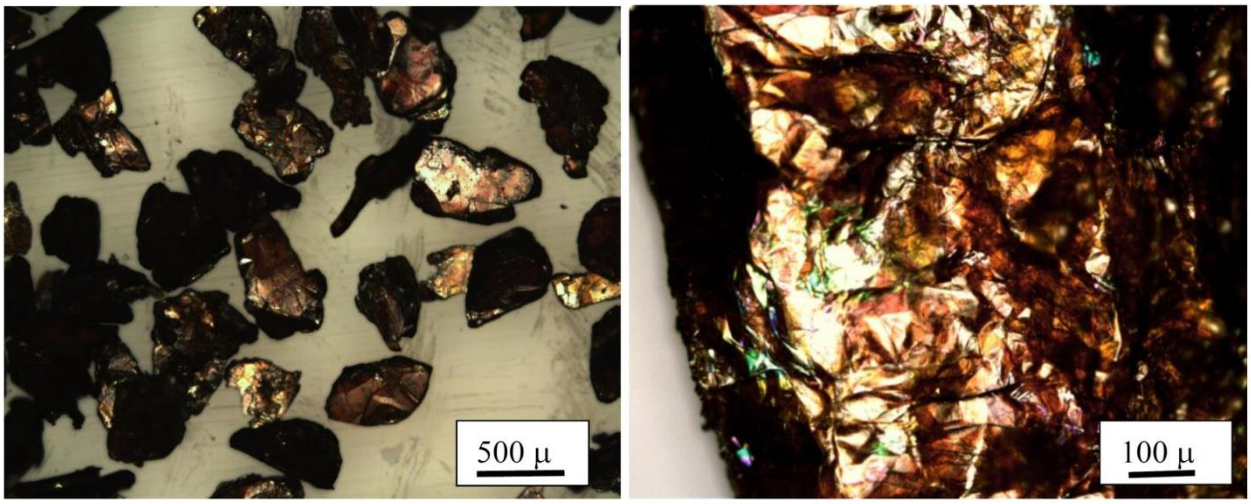

Figure S1. Optical images of fluorinated graphite crystallites with different magnification.

Fluorinated graphite intercalation compounds with different stoichiometry of matrix and different guest molecules were synthesized using a procedure described in Refs[1-4]. At the first step, graphite crystallites were placed in a Teflon flask and held there in the vapors over liquid Br_2 for 2 days. The resultant bromine-intercalated graphite was transferred into another Teflon flask and located over a solution of BrF_3 in Br_2. An amount of BrF_3 in the solution corresponded to the planned stoichiometry of graphite fluorides C_2F_x ($x \approx 0.5, 0.8, 1.0$). The fluorination process was conducted at room temperature and it required about 1 month for the samples with a high fluorine content and up to 3 months for the samples with a reduced content of fluorine. The fresh samples, prepared by this method, contained the remains of oxidizing media between matrix layers and this media was immediately substituted into pure Br_2 and then to pure CH_3CN[2]. Then, CH_3CN was replaced by another guest, such as $(CH_3)_2CO$, CH_2Cl_2, $C_2H_4Cl_2$ etc., by putting the sample in corresponding organic liquid. After drying in N_2 gas, some of the solvent molecules leave the sample, and the filling of the layers becomes irregular, forming a mixed-stage structure with a predominantly bilayer character[5].

According to atomic emission spectral (AES) analysis, the content of Fe, Co, Ni in purified graphite and fluorinated graphite samples was $1 \cdot 10^{-3}$, $<1 \cdot 10^{-4}$, and $<5 \cdot 10^{-5}$ wt.%, respectively.

2. Characterization

The structure and composition of the fluorinated graphite samples were studied by means of X-ray diffraction (XRD) on a DRON-SEIFERT-RM4 diffractometer using CuK_α radiation and X-ray photoelectron spectroscopy (XPS) on a Phoibos 150 SPECS spectrometer using a monochromatized AlK_α radiation with the energy of 1486.7 eV.

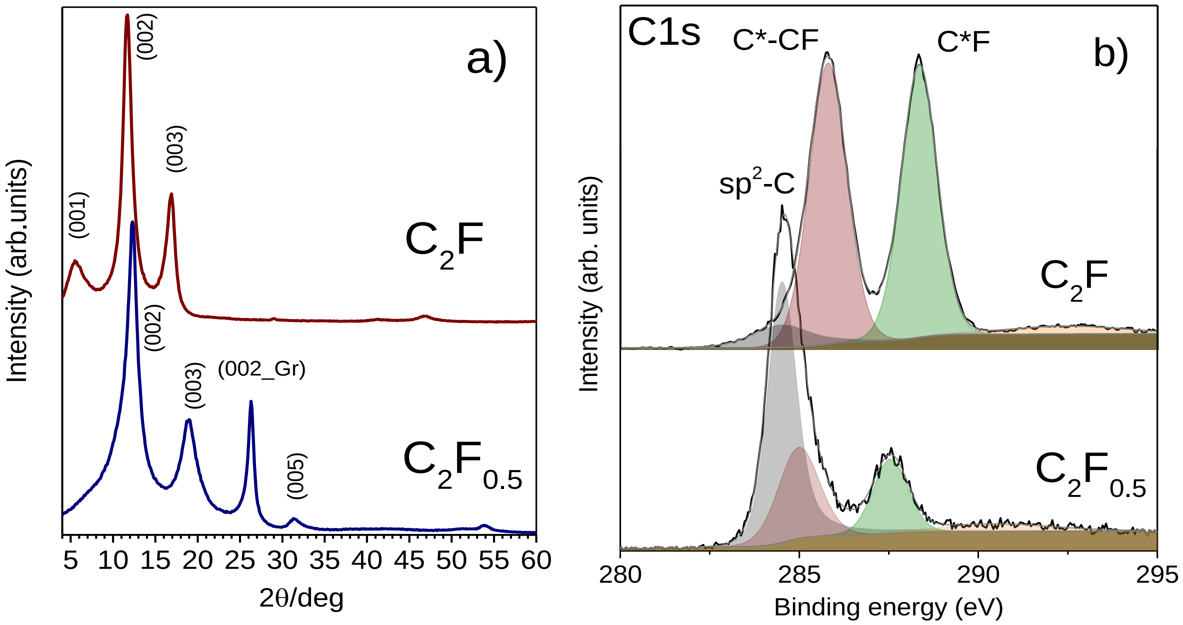

Figure S2. X-ray diffraction patterns **(a)** and X-ray photoelectron C 1s spectra **(b)** of acetonitrile-intercalated fluorinated graphite with matrix compositions of C_2F and $C_2F_{0.5}$.

The results of XRD analysis of acetonitrile-intercalated C_2F and $C_2F_{0.5}$ samples are shown in Fig. S2(a). The XRD pattern of C_2F sample has a set of *00l* reflections at 2Θ equal to 5.6° (*001),* 11.6° (*002*), and 16.9° (*003*). The calculated identity period along the *c* axis of this sample is 15.5 (±0.2) Å, that is smaller than 16.1 Å obtained by simple summation of double thickness of empty C_2F matrix (6.05 Å) and the width of CH_3CN molecule (≈ 4.0 Å). This is due to the close packing of guest molecules into interlayer spacing[6]. The XRD data indicates that the fluorinated graphite intercalation compounds with a composition of matrix of C_2F_x, $x \approx 1$, is second-stage intercalates. The pattern of $C_2F_{0.5}$ sample has reflections from two subsystems. The interlayer distances 7.20 Å (*002*, 12.3°), 4.72 Å (*003*, 18.9°), 2.86 Å (*005*, 31.3°) correspond to the fluorinated graphite intercalation compound with the identity period of 14.2 (±0.2) Å. The *(002)* reflection at 2Θ = 26.3° originates from graphite-like regions. An absence of the *(001)* reflection from the fluorinated graphite matrix and a broadening of other *00l* reflections are the sign of lower structural ordering of the $C_2F_{0.5}$ sample as compared to the C_2F sample.

XPS C 1s spectra of the C_2F sample showed two peaks corresponding to the fluorinated carbon atoms (C*F component) and bare carbon atoms linked with CF groups (C-C*F component)

(Fig. S2b). The presence of two peaks with similar intensities demonstrates the propriety of C_2F structure as alternation of conjugated carbon bond chains and of covalently bonded (C–F) carbon atoms chains. The C 1s spectrum of $C_2F_{0.5}$ sample has a dominant peak at 284.5 eV characteristics of the sp^2-hybridized carbon. The areas constituted from these carbon atoms separate the fluorinated areas in the graphene planes.

3. Reproducibility: samples with different intercalants

During our studies of fluorinated graphite, we have measured about 20 samples with various fluorine content and different intercalant molecules such as acetonitrile CH_3CN, dichloromethane CH_2Cl_2, dicloroethane $C_2H_4Cl_2$, and acetone $(CH_3)_2CO$. Excepting the difference in chemical composition, the samples were synthesized and then aged in identical conditions.

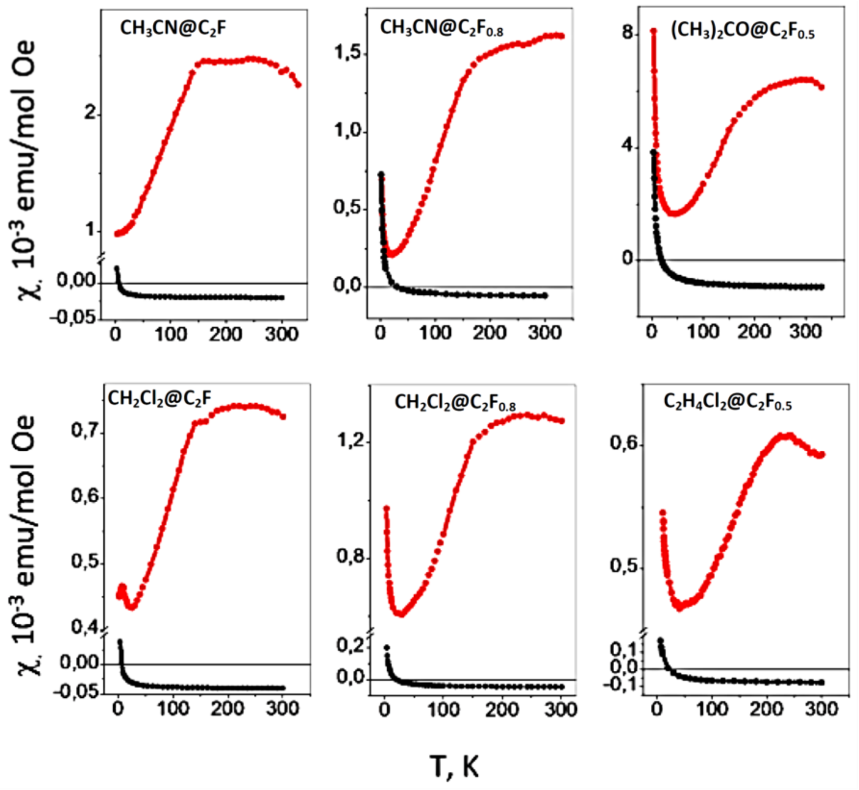

Figure S3. Temperature dependencies of the magnetic susceptibility of fluorinated graphite samples with different matrix composition (C_2F_x) and different intercalated molecules (acetonitrile CH_3CN, dichloromethane CH_2Cl_2, dicloroethane $C_2H_4Cl_2$, and acetone $(CH_3)_2CO$). The black curves correspond to the fresh samples, the red curved were obtained for the same samples after one-year storage.

Majority of the samples reproduced magnetic features similar to that described in the manuscript, the most prominent of which is that the aging changed magnetic response from essentially diamagnetic to non-Curie paramagnetic. The temperature dependence of the magnetic susceptibility for some typical samples, as prepared and aged, is shown in Figure S3. Irrespective

the intercalant molecule nature, the overall magnetic features are reproduced, and these data support our main result that the nontrivial magnetism of fluorinated graphite originates from the *Tabby* fluorinated carbon planes.

References:

1. Yudanov, N. F., Ukraintseva, E. A., Chernyavsky, L. I., Yakovlev, I. I. Intercalate Vapor Pressure over Graphite Fluoride Intercalation Compounds with Acetonitrile. *Izv. SO AN SSSR, Ser. Khim. Nauk*. **3**, 30-34 (1989).
2. Pinakov, D. & Logvinenko, V. The relationship between properties of fluorinated graphite intercalates and matrix composition. *J. Therm. Anal. Calorim.* **86**, 173-178 (2006).
3. Pinakov, D., Logvinenko, V., Shubin, Y. & Chekhova, G. The relationship between properties of fluorinated graphite intercalates and matrix composition: Part II. Intercalates with chloroform. *J. Therm. Anal. Calorim.* **90**, 399-405 (2007).
4. Chekhova, G., Pinakov, D., Shubin, Y. V. & Logvinenko, V. Structural rearrangements of the first stage inclusion compound of fluorinated graphite with acetonitrile during isothermal deintercalation. *J. Therm. Anal. Calorim.* **128**, 349-355 (2016).
5. Yudanov, N., Chernyavskii, L., Lisoivan, V. & Yakovlev, I. Structure of intercalated compounds of graphite fluoride C_2F_X. *J. Struct. Chem.* **29**, 412-418 (1988).
6. Chekhova, G.N., Ukraintseva, E.A., Ivanov, I.M., Yudanov, N.F., Shubin, Yu.V., Logvinenko, V.A. et al. Influence of the matrix composition on the properties of fluorinated graphite inclusion compounds with acetonitrile. *Russ. J. Inorg. Chem.* **50**, 1055-1061 (2005).
